# Thyroid stimulating hormone levels in cord blood are not influenced by non-thyroidal mothers’ diseases

**DOI:** 10.1590/S1516-31802000000500006

**Published:** 2000-09-01

**Authors:** Laura Sterian Ward, Ilda Shizue Kunii, Rui Monteiro de Barros Maciel

**Keywords:** Congenital hypothyroidism, Screening program, Maternal diseases, Hipotiroidismo congênito, Rastreamento, Doença materna

## Abstract

**CONTEXT:**

Screening programs not only offer the opportunity to trace and treat almost all cases of congenital hypothyroidism but also mean large savings to the health system. However, carefully planned strategies are necessary to extend their benefits and reduce costs.

**OBJECTIVE:**

To determine the possible influence of maternal diseases that affect maternal-fetal placenta dynamics on primary thyroid stimulating hormone (TSH) screening for congenital hypothyroidism.

**DESIGN:**

Prospective non-randomized clinical trial with at least 3 months of follow-up.

**SETTING:**

A public university referral center [CAISM/Hospital das Clínicas, Faculty of Medicine, University of Campinas, Campinas, SP].

**PARTICIPANTS:**

415 neonates divided into 5 groups: eighty-three infants born from cardiac mothers; 98 from mothers that had toxemia; 54 of the mothers had diabetes mellitus; 40 were HIV positive and 140 had no diseases.

**INTERVENTION:**

All newborns had cord blood samples collected on filter paper at birth.

**MAIN MEASUREMENTS:**

TSH was measured from dried blood spots using a homemade immunofluorescence assay (sensitivity in dried blood spots = 0.1 mU/L).

**RESULTS:**

There was no significant difference in the mean TSH levels among the 5 groups. Moreover, TSH levels were around 5 mU/L in 48% of the newborns, indicating that our region is severely deficient in iodine.

**CONCLUSIONS:**

Our results indicate that primary TSH screening programs using cord blood are not affected by maternal diseases. We suggest that, besides its technical advantages over heel punctures with T_4_ primary approaches, neonatal screening using primary cord blood TSH may also be used as a monitoring tool for evaluation and control of iodine deficiency disorders (IDD).

## RESUMO

**CONTEXTO:**

Os programas de detecção precoce trazem economias ao sistema de saúde e oferecem a oportunidade de rastrear e tratar precocemente casos de hipotiroidismo congênito.

**OBJETIVO:**

Determinar influências de doenças que afetam a dinâmica materno-fetal-placentária sobre programas de detecção precoce de hipotiroidismo congênito que se baseiam na dosagem do hormônio tirotrófico (TSH).

**TIPO DE ESTUDO:**

Ensaio clínico prospectivo não-randomizado com, ao menos, três meses de seguimento.

**LOCAL:**

Centro Universitário Público de Referência - Centro de Atendimento Integrado a Saúde da Mulher (CAISM).

**PARTICIPANTES:**

415 recém-nascidos de 5 grupos de parturientes: 83 crianças eram filhas de mães cardiopatas; 98 de mães com toxemia gravídica; 54 de mães diabéticas; 40 de mães portadoras de imunodeficiência adquirida (HIV); e 140 de mães hígidas.

**PROCEDIMENTOS:**

Todos os recém-nascidos tiveram amostras de sangue de cordão umbilical coletadas em papel de filtro ao nascimento.

**VARIÁVEIS ESTUDADAS:**

Dosagem de TSH em sangue coletado em papel de filtro usando um ensaio imunofluorométrico próprio (sensibilidade em manchas de sangue seco = 0.1 mU/L).

**RESULTADOS:**

Não encontramos diferença na média de TSH dos 5 grupos. Além disso, os níveis de TSH estavam acima de 5 mU/L em 48% dos bebês, sugerindo que nossa região é severamente deficiente em iodo.

**CONCLUSÕES:**

Nossos resultados demonstram que programas de detecção precoce de hipotiroidismo congênito, que utilizam primariamente TSH, não são afetados por doenças maternas não-tiroidianas. Sugerimos que, além das vantagens técnicas sobre a punção de calcanhar com dosagem primária de T_4_, os programas de detecção precoce que utilizam primariamente TSH de cordão umbilical também podem ser usados como instrumento de avaliação e controle da carências de iodo.

## INTRODUCTION

Systematic neonatal screening has been progressively implemented in industrialized countries over the past 2 decades. Screening programs for congenital hypothyroidism (CH) have also been expanding throughout South America and developing countries, where severe mental deficiency from CH has clearly been reduced.^1^ The vast majority of programs are now using a primary thyroxine (T_4_)-backup thyrotrophin (TSH) or a primary TSH test.^2^ In Brazil, cord blood evaluation has been recommended, particularly because of the short hospital stay of our normal pregnant women.^3^ Also, TSH has been recommended as the primary screening test because it detects not only permanent sporadic congenital hypothyroidism, whose incidence is about 1 per 3500-4000 births in our region, but also compensated or transient primary hypothyroidism, whose incidence can be as high as 1 in 10 neonates and whose main cause is iodine deficiency.^3–6^ There is some evidence that our region is deficient in iodine supplementation.^7,8^ However, T_4_ primary screening programs have still been carried out and are widely preferred in many assistance centers in Brazil. Although there have been some previous studies on the perinatal factors influencing TSH and T_4_ concentrations in cord blood, particularly in high-risk newborns, those small for gestational age and/or preterm infants, there is still poor knowledge about the possible influence of severe maternal diseases over sensitive TSH measurement methods. The aim of the present work was to investigate the influence of nonthyroidal mothers’ diseases on our routine screening program using primary cord TSH values.

## METHODS

The procedures that follow were in accordance with the ethical standards of the committee responsible for human experimentation and with the Helsinki declaration of 1975, as revised in 1983.

### Setting

A public university referral center, the Center for Integrated Assistance in Women's Health at the University of Campinas - Centro de Assistência Integrada à Saúde da Mulher/Hospital das Clínicas da Faculdade de Medicina da Universidade de Campinas, Campinas.

### Design

Prospective non-randomized clinical trial with at least 3 months follow-up.

### Participants

We examined and enrolled a minimum of 415 newborns from the Maternity Department of the University of Campinas (CAISM/UNICAMP). In accordance with their mothers’ profiles, they were divided into 5 groups:

*Group A.* Composed of 83 infants of mothers with congestive cardiac diseases, including Chagas’ disease, hypertensive cardiac disease, and valve disease. They were divided into Functional Type II (n = 23 patients), Functional Type III (n = 39) and Functional Type IV (n = 21), according to the criteria committee of the New York Heart Association.^9^

*Group B.* Composed of 98 infants from mothers with toxemic pregnancies classified as moderate (diastolic blood pressure < 10 mmHg; n = 72 patients) or severe (diastolic blood pressure > 10 mmHg; n = 26 patients).

*Group C.* Composed of 54 infants from diabetic mothers, classified into type A (n = 30), type B (n = 13) and type C (n = 11), according to the Priscilla White classification.^10^

*Group D.* Composed of 40 infants from mothers infected by the human immunodeficiency virus (HIV positive) but with no active disease.

*Group E.* A control group of 140 normal pregnant women.

### Diagnostic test

Cord blood samples were collected by labor-room personnel. TSH concentrations were measured in 2-3 out of 5 cord blood spots collected on filter paper, using a homemade immunofluorescence assay described elsewhere.^11^ The detection limit of the assay in dried blood spots is 0.1 mU/L and in plasma is 0.05 mU/L, with inter and intra-assay coefficients of variation of 5 and 10%, respectively. T_4_ was also measured with a fluorometric assay with a sensitivity of 1.6 mg/dL, with inter and intra-assay coefficients of variation of 5 and 10%, respectively.

### Statistical Methods

Computer statistical programs, EPIINFO and STATGRAG, Microsoft 5.0, were used for analyzing data. Duncan, Ryan-Einot-Gabriel Welsh, chi-square (χ^2^), Kruskal-Wallis (H) and ANOVA (F) tests were used to verify differences between groups and subgroups. Results were expressed as the mean ± standard deviation (SD). The level of significance was taken as P < 0.05.

## RESULTS

**Figure 1:**
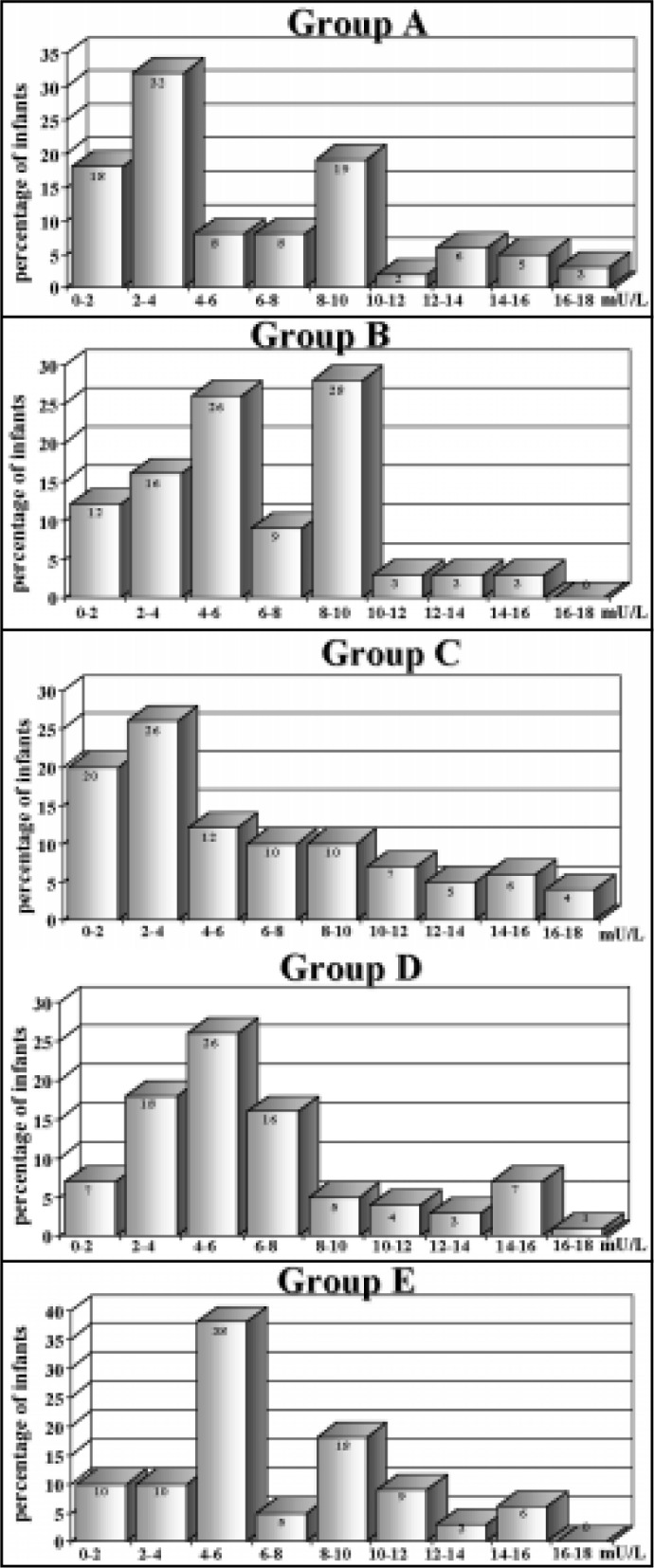
Percentile distribution of TSH levels for the 5 different groups of infants born from: group A: toxemic mothers (N = 98); group B: diabetic mothers N = 54); group C: cardiac disease mothers (N = 83); group D: HIV positive mothers and group E: control healthy mothers (N = 140). P > 0.05.

The infant's sex, duration of labor, mode of delivery and use of uterogenic agents were similar in all groups (χ^2^ = NS). Table 1 represents TSH mean levels, expressed as the mean and standard deviation, in patients of the 5 groups. In Figure 1, these data are expressed as their percentile distribution in each of the 5 groups.

**Table 1:** Thyroid stimulating hormone (TSH) levels in mU/L, expressed as mean and standard deviation, of infants of group A: cardiac disease mothers (n = 83); group B: toxemic mothers (n = 98); group C: diabetic mothers (n = 54); group D: HIV positive mothers; group E: control healthy mothers (n = 140). Group C is subdivided into Priscilla White classes A (n = 30), B (n = 13) and C (n = 11). Statistical comparison revealed no difference among groups (P > 0.05)

Groups	Thyroid stimulating hormone	
	Mean	SD
A	6.51	3.58
B	6.13	4.85
C	6.08	4.76
Class A	6.43	4.58
Class B	6.94	3.98
Class C	6.04	4.15
D	6.42	4.10
E	6.42	4.10

Comparison of these results shows no statistical difference among groups or subgroups (H = 0.4963; P = 0.29; F = 0.609, P = 0.65). All values were below our cut-off value for congenital hypothyroidism (20 mU/L). No child demonstrated any feature that could be suspected as congenital hypothyroidism during a follow-up of, at least, 3 months (mean 5 months, SD 2). Forty-eight percent of the newborns presented a TSH cord blood level above 5 mU/L. There was no statistical difference in the distribution of TSH > 5 mU/L cases in the different groups.

## DISCUSSION

Clinical diagnosis of congenital hypothyroidism is difficult at birth and measurements of TSH are essential for this diagnosis. A screening program not only offers the opportunity to trace and treat almost all cases but also means large savings to the country.^12^ However, to extend the benefits of screening programs and reduce costs, carefully planned strategies are necessary. The Brazilian Endocrine Society recommends a primary TSH, T_4_ back-up program on cord blood samples. However, T_4_ has still been defended as the ideal method by many services that also use heel puncture performed at the 3rd to 5th day of life. We have previously demonstrated that, although both strategies are able to detect all cases of hypothyroidism, umbilical cord blood is technically superior to heel puncture and primary TSH clearly reduces the recall index for confirmation of the results.^13^

Infants born prior to term have lower cord serum T_4_ concentrations that correlate with gestational age or birth weight.^14^ Previous reports have shown that other factors, like maternal diseases affecting placental dynamics, may influence T_4_ values and thus the screening programs that use primary T_4_.^15,16^ However, data on the possible influence of these factors on cord blood TSH are scarce. Franklin, et al. analyzed the effects of maternal diabetes mellitus, toxemia, fetal distress and other factors and concluded that they did not affect cord serum TSH concentrations.^15^ Only the delivery method influenced cord serum T_4_. Another early report from Fuse, et al. also suggested that TSH values in cord blood could be less influenced by perinatal factors than T_4_ values.^16^ We investigated newborns from mothers affected by severe non-thyroidal diseases, including HIV. Our data confirm that even severe maternal diseases do not affect a screening program using primary TSH from cord blood.

The World Health Organization (WHO), United Nations International Children's Emergency Fund (UNICEF), and the International Council for Control of Iodine Deficiency Disorders (ICCIDD) have included neonatal TSH as one of the indicators for assessing iodine deficiency disorders (IDD) and their control.^6^ In the absence of iodine deficiency, the frequency of neonatal TSH above 5 mU/L whole blood (or 10 mU/L serum) is less than 3%. A frequency of 3-19.9% indicates mild IDD. Frequencies of 20-39.9% and above 40% indicate moderate and severe IDD, respectively. We found a 48% prevalence of TSH cases above 5 mU/L indicating that our region is severely deficient in iodine. Recently Haddow et al suggested that maternal thyroid dysfunction during pregnancy may impair the subsequent neuropsychological development in children, raising our concerns about iodine supplementation.^8,17^

We suggest that, besides its technical advantages over heel punctures with T_4_ primary approaches, neonatal screening using primary cord blood TSH may also be used as a monitoring tool for IDD evaluation and control in Brazil.
